# Erweiterte präoperative sprachaudiometrische Diagnostik im Rahmen der Cochleaimplantatversorgung

**DOI:** 10.1007/s00106-023-01344-4

**Published:** 2023-08-15

**Authors:** Annika Beyer, Jan-Henrik Rieck, Alexander Mewes, Jan Andreas Dambon, Matthias Hey

**Affiliations:** 1 Klinik für Hals-Nasen-Ohren-Heilkunde, Kopf- und Halschirurgie, Universitätsklinikum Schleswig-Holstein (UKSH), Campus Kiel, Kiel, Deutschland; 2 Medizinische Fakultät, der Christian-Albrechts-Universität zu Kiel, Kiel, Deutschland

**Keywords:** Freiburger Einsilbertest, Wortverstehen, Hörgerät, Präoperative Messungen, CI-Leitlinie, Freiburg monosyllablic test, Word recognition, Hearing aid, Preoperative measurements, CI guideline

## Abstract

**Hintergrund:**

Bei einer hochgradigen, bis an Taubheit grenzenden Schallempfindungsschwerhörigkeit ist das Cochleaimplantat (CI) die Therapie der Wahl. Für die Indikationsstellung zur Cochleaimplantation sind das präoperativ gemessene Freiburger Einsilberverstehen (EV) bei 65 dB SPL mit Hörgerät (HG) im Freifeld (EV_HG_65) und das maximale Einsilberverstehen ohne HG mit Kopfhörern (mEV) von entscheidender Bedeutung. Ziel dieser retrospektiven Studie war die Analyse des Zusammenhangs zwischen EV mit dem HG bei 80 dB SPL (EV_HG_80) und dem mEV. Dies ist eine Ergänzung zur Bestimmung des EV_HG_65 im Vergleich zum mEV und zum Reintonaudiogramm (4FPTA).

**Material und Methodik:**

Im Rahmen dieser Studie wurde das EV mit und ohne HG bei 661 Ohren retrospektiv analysiert. Einschlusskriterium war die spätere Implantation mit einem CI.

**Ergebnisse:**

Im Rahmen der präoperativen CI-Diagnostik ergibt sich ein mEV von 0 % bei 334 Ohren. Ebenso lag das EV_HG_65 bei 485 Ohren bzw. das EV_HG_80 bei 335 Ohren bei 0 %. Das EV mit HG verschlechterte sich mit zunehmendem 4FPTA für beide Prüfpegel, wobei der Effekt bei 80 dB SPL weniger stark ausgeprägt war als bei 65 dB SPL. Werden nur Ohren betrachtet, welche präoperativ ein mEV > 0 % hatten (*n* = 260), zeigt sich eine stärkere Übereinstimmung zwischen dem EV_HG_80 und dem mEV mit einer Differenz von (−4,0 ± 16,4 %) im Vergleich zum EV_HG_65 und dem mEV mit einer Differenz von (−18,3 ± 16,7 %). Es zeigt sich ein signifikanter Unterschied zwischen dem mEV und dem EV_HG_80 im Vergleich zum mEV und dem EV_HG_65.

**Schlussfolgerung:**

Das EV mit HG zeigt bei einem Sprachpegel von 65 dB SPL oftmals nicht die nach Hilfsmittelrichtlinie und CI-Leitlinie geforderte Annäherung des EV an das mEV. Das EV_HG_80 zeigt eine bessere Deckung mit dem mEV als das EV_HG_65. Es ist in der klinischen Diagnostik sinnvoll, das Sprachverstehen mit HG ergänzend oberhalb von 65 dB SPL zu untersuchen.

## Abstract (en)

**Background:**

For severe hearing loss and even profound deafness, cochlear implants (CIs) have become the treatment of choice. For establishment of the CI indication, the preoperative Freiburger monosyllabic word recognition (EV) at 65 dB SPL in free field with a hearing aid (EV_HG_65) and the maximal understanding (mEV) without a hearing aid with headphones results are important. The goal of this retrospective study was to analyse the correlation of word recognition with a hearing aid at 80 dB SPL (EV_HG_80) and mEV. This represents an extension to measuring EV_HG_65 compared to mEV and to pure-tone audiometry (4FPTA).

**Methods:**

In this study, word recognition with and without a hearing aid was retrospectively analysed for 661 ears. Inclusion criterium was CI implantation at a later date.

**Results:**

During preoperative CI diagnostics, an mEV of 0% was found in 334 ears. The EV_HG_65 for 485 ears and the EV_HG_80 for 335 ears were also 0%. The EV with hearing aid was found to worsen with increasing 4FPTA at both sound pressure levels, although this effect was smaller at 80 dB SPL than at 65 dB SPL. Including only ears with mEV > 0 % (*N* = 260 ears), a stronger correlation between EV_HG_80 and mEV with a difference of (−4.0 ± 16.4%) in comparison to EV_HG_65 and mEV with a difference of (−18.3 ± 16.7%) is seen. This shows a significant difference between mEV and EV_HG_80 compared to mEV and EV_HG_65.

**Conclusion:**

At a sound pressure level of 65 dB SPL, EV with hearing aid often does not show the accordance with mEV specified by hearing aid and CI guidelines. The EV_HG_80 correlates better with mEV than EV_HG_65. For clinical diagnosis it is rational to measure speech discrimination with hearing aid at levels higher than 65 dB SPL.

Bei einer hochgradigen an Taubheit grenzenden Schallempfindungsschwerhörigkeit ist heutzutage das Cochleaimplantat (CI) die Therapie der Wahl [1, 12]. Während zu Beginn der CI-Versorgung ein CI fast ausschließlich bei PatientInnen implantiert wurde, die weder mit noch ohne konventionelle Hörhilfe ein Sprachverstehen erlangten [11], haben sich die Indikationsgrenzen aufgrund des großen Therapieerfolgs sowie der technischen Weiterentwicklung der Implantate, Elektroden und Sprachprozessoren inzwischen soweit verschoben, dass auch PatientInnen mit Restgehör und Restverstehen [9, 16, 19] sowie mit einseitiger Ertaubung („single-sided deafness“, SSD) [2] versorgt werden. Die Qualitätssicherung bei der Indikationsstellung für ein CI erfordert eine strukturierte präoperative Diagnostik, die sich an den unterschiedlichen individuellen Voraussetzungen zur Cochleaimplantation orientiert. Zur Unterstützung der Entscheidungsfindung steht in Deutschland die S2k-Leitlinie zur CI-Versorgung [1] zur Verfügung, die, neben der etablierten Tonaudiometrie, vor der CI-Operation auch Untersuchungen zum Sprachverstehen mit und ohne Hörgerät empfiehlt. Die präoperative Messung des Sprachverstehens in Ruhe erfolgt unter Verwendung des Freiburger Sprachverständlichkeitstests [6]. Ziel ist hierbei die Ermittlung des maximalen Einsilberverstehens (mEV) und des Hörverlusts für Zahlwörter ohne Hörgerät über Kopfhörer durch die pegelabhängige Messung des Einsilberverstehens. Zur Absicherung der bestmöglichen konventionellen apparativen Versorgung erfolgt zusätzlich eine sprachaudiometrische monaurale Kontrolle des Einsilberverstehens mit Hörgerät (HG) im Freifeld. Dabei wird regelhaft ein Sprachpegel von 65 dB SPL verwendet [1, 3]. Eine Hörgeräteversorgung wird als zweckmäßig angesehen, wenn das bei 65 dB SPL gemessene Einsilberverstehen mit dem HG (EV_HG_65) um mind. 20 Prozentpunkte besser ausfällt als ohne HG und dieses Verstehen mit dem Hörgerät dem mEV „möglichst nahe“ kommt [3, 18].

Mit Blick auf die Beziehung von Einsilberverstehen mit HG und mEV zeigten Hoppe et al. [9, 10], dass das EV_HG_65 meist 10–20 Prozentpunkte unterhalb des mEV liegt. Des Weiteren war nur bei 40 % der betrachteten Hörgeräteträger die Differenz zwischen mEV und dem Verstehen mit HG kleiner als 10 %. Vergleichbare Ergebnisse fanden Müller et al. im Rahmen einer altersabhängigen Betrachtung [14], wobei die Diskrepanz zwischen mEV und EV_HG_65 vor allem bei PatientInnen mit hochgradigen bzw. hochgradigen bis an Taubheit grenzenden Hörverlusten und höherem Alter beobachtet wurde [10]. Die geringe Deckung von mEV und EV_HG_65 kann durch die mangelhafte Verstärkung der hochverstärkenden Hörgeräte bei diesem Pegel erklärt werden [5]. Um das Verstehen unter Ausschöpfung des gesamten Dynamikbereichs zu betrachten, sollte das Einsilberverstehen grundsätzlich auch bei Sprachpegeln unter- und oberhalb von 65 dB SPL untersucht werden, wie es in der CI-Leitlinie empfohlen wird [1].

Das Ziel dieser retrospektiven Studie war es, das EV mit HG sowohl bei 65 dB SPL als auch bei 80 dB SPL dem mEV gegenüberzustellen. Dafür erfolgte die Kontrolle des Verstehens mit HG bei 65 dB SPL und bei 80 dB SPL mit HG (EV_HG_80). Zudem wurde analysiert, ob das präoperativ über Kopfhörer gemessene Tonhörvermögen einen Einfluss auf das mEV sowie das Verstehen mit HG aufweist.

## Methodik

### PatientInnen

Bei dieser retrospektiven Studie wurden die Daten von 661 Ohren bei 576 PatientInnen (491 unilateral und 85 bilateral versorgt), die im Rahmen der präoperativen CI-Diagnostik an der Klinik für Hals‑, Nasen‑, Ohrenheilkunde, Kopf- und Halschirurgie des Universitätsklinikums Schleswig-Holstein (UKSH) am Campus Kiel im Zeitraum 2002 bis 2019 vorstellig wurden, anonymisiert ausgewertet. Es wurden nur PatientInnen eingeschlossen, welche muttersprachlich Deutsch sprachen und zum Zeitpunkt der Implantation mindestens 18 Jahre alt waren. Das mittlere Alter aller PatientInnen war 56 ± 18 Jahre mit einer Spannweite von 20–89 Jahren. Die Studie wurde vorab durch die lokale Ethikkommission genehmigt (EK der Medizinischen Fakultät, Universität Kiel, D 455/20). Aufgrund des retrospektiven Studiendesigns waren nicht für alle Ohren alle Daten vollständig, sodass in den unterschiedlichen Auswertungen teilweise Ohren ausgeschlossen werden mussten und sich dadurch die PatientInnenzahl reduzierte. Die konkrete Anzahl wird für jede Untersuchung angegeben.

### Ton- und Sprachaudiometrie

Im Rahmen dieser Studie wurden folgende audiometrische Kenndaten retrospektiv analysiert:

- *4FPTA* („four-frequency pure-tone average“): Mittelwert der tonaudiometrischen Hörschwelle der Frequenzen 500, 1000, 2000 und 4000 Hz. Die Darbietung der Testtöne erfolgte monaural über Kopfhörer, wobei die Gegenseite bei Bedarf vertäubt wurde [13]. Frequenzen, bei denen bis 120 dB HL keine tonaudiometrische Schwelle ermittelt werden konnte, wurde der Wert 130 dB HL zugeschrieben. Daraus ergab sich, dass einige PatientInnen einen 4FPTA von bis zu 130 dB HL haben.
- *mEV*: maximales Einsilberverstehen, monaural über Kopfhörer (TDH-39P der Fa. Telephonics Corporation, New York, USA) ohne HG gemessen. Das Einsilberverstehen wurde beginnend bei 65 dB SPL mit ansteigendem Schalldruckpegel in 15-dB-Schritten bis an den Pegel des mEV bzw. bis an die Unbehaglichkeitsschwelle gemessen. Der maximale Testpegel lag bei 120 dB SPL. Als Sprachmaterial wurde der Freiburger Einsilbertest nach Hahlbrock [6] entsprechend der DIN-Norm 45621‑1 [4] genutzt. Dieses wurde zur Minimierung des repetitiven Lerneffekts nach zwei Verfahren randomisiert: Zum einen wurde die Reihenfolge der Testlisten an einem Termin randomisiert, um nicht die gleichen Listen wie bei den vorherigen Untersuchungen zu nutzen. Zum Zweiten wurde die Reihenfolge der Wörter innerhalb einer Liste stets neu randomisiert. Es wurden alle 20 Testlisten genutzt.
- *EV*_*HG*_*65* und *EV*_*HG*_*80*: Verstehen von Einsilbern aus dem Freiburger Sprachverständlichkeitstest mit HG, seitengetrennt gemessen über einen 1,3 m frontal zur PatientIn aufgestellten Lautsprecher. Bei einigen PatientInnen lagen aufgrund von Optimierungen der HG mehrere Messungen vor. In diesem Fall wurde dabei stets das bestmögliche Verstehen analysiert, d. h. diejenige Messung verwendet, bei der das EV_HG_65 möglichst nahe am mEV liegt. Die Randomisierung der Testlisten erfolgte analog zum mEV.

Bei asymmetrischen Hörverlusten wurde das bessere Ohr stets vertäubt [13].

Für die Untersuchungen kam das Audiometriemesssystem evidENT 3.0 (Fa. Merz Medizintechnik; Reutlingen, Deutschland) mit einem Audiometer der Fa. Interacoustics (Middlefart, Dänemark) zum Einsatz. Die Messungen erfolgten in schallisolierten Hörprüfkabinen (Maße = 6 × 4 × 2,5 m^3^).

### Statistik

Die statistische Auswertung erfolgte mittels IBM SPSS Statistics 28.0.0.0. Es wurde untersucht, ob der 4FPTA und das mEV einen Einfluss auf das EV_HG_65 und EV_HG_80 haben und ob das mEV vom individuellen 4FPTA abhängt. Zur Veranschaulichung der Zusammenhänge zwischen dem individuellen Hörverlust und dem EV mit HG sowie dem mEV wurden die Streudiagramme visuell inspiziert und der Spearman-Korrelationskoeffizient (Rho) bestimmt. Die von Holube, Winkler und Nolte-Holube [7] ermittelten 90%-Konfidenzintervalle für die Test-Retest-Genauigkeit des Freiburger Einsilbertests bei Schwerhörigen wurden herangezogen, um das Einsilberverstehen mit HG im Vergleich zum mEV unter Berücksichtigung der Messgenauigkeit des Freiburger Einsilbertests zu analysieren. Boxplots wurden erstellt, um die Differenzen zwischen dem mEV und dem EV_HG_65 und EV_HG_80 grafisch darzustellen. Es wurden der Median, das erste und dritte Quartil, die Whisker und Ausreißer abgebildet. Die Whisker der Boxplots wurden als das 1,5-Fache des Interquartilbereichs definiert. Vor der statistischen Auswertung wurden die Daten auf Normalverteilung getestet. Die Prüfung auf Normalverteilung erfolgte mithilfe des Kolmogorov-Smirnov-Tests und des Shapiro-Wilk-Tests. Die Tests zeigten keine Normalverteilung. Bei der weiterführenden Betrachtung der Verteilungsfunktion und der Daten zeigte sich eine glockenförmige Verteilung. Das Q‑Q-Diagramm zeigte ebenfalls eine Normalverteilung. Die Diskrepanz zwischen der Testung auf Normalverteilung und der grafischen Beurteilung könnte daraus resultieren, dass das mEV = 0 % bei der grafischen Beurteilung vernachlässigt wurde, nicht aber bei der Testung auf Normalverteilung. Aufgrund dessen wurden bei allen Daten nichtparametrische Tests wie der Spearman-Korrelationskoeffizient (Rho) und der Wilcoxon-Test genutzt.

## Ergebnisse

**Figure 1:**
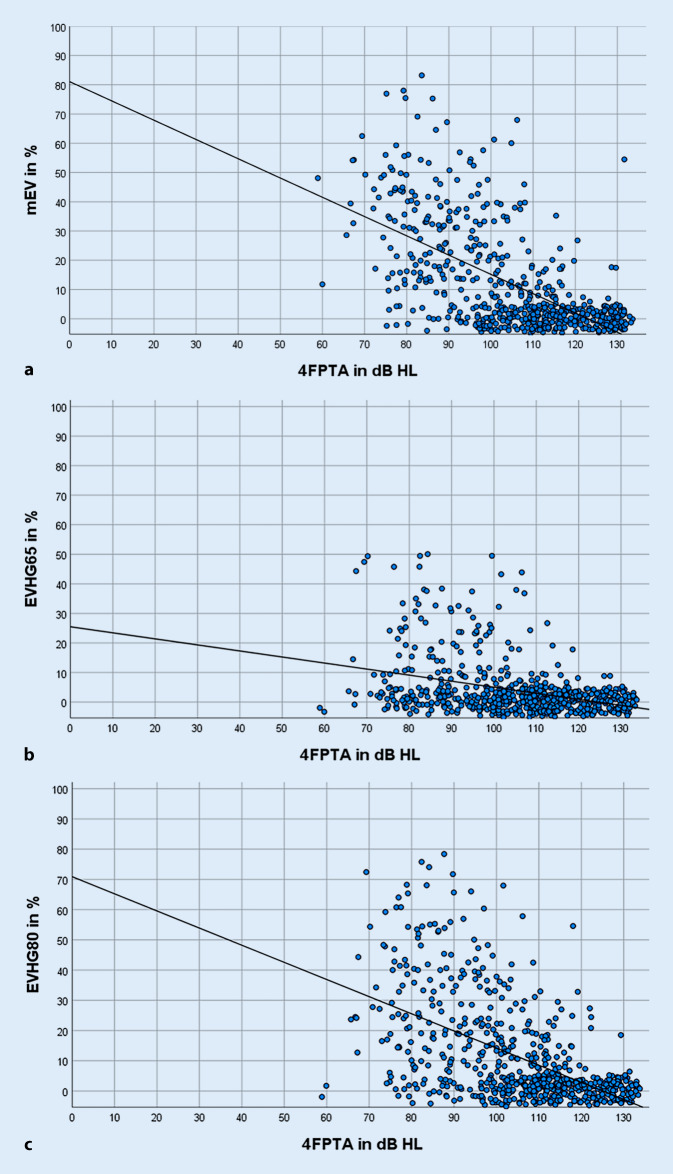

Bei allen 661 Ohren konnten sowohl das mEV als auch das EV_HG_65 und EV_HG_80 aus den vorliegenden Messungen retrospektiv ermittelt werden. In Abb. 1 ist das in den drei Messkonditionen (mEV, EV_HG_65 und EV_HG_80) registrierte Einsilberverstehen in Abhängigkeit vom 4FPTA dargestellt. Es ist zu erkennen, dass das mEV mit zunehmendem Hörverlust abnimmt (Abb. 1a). Die Werte des maximalen Einsilberverstehens verteilten sich im Bereich von 0 bis 85 %, wobei sich bei 334 Ohren ein mEV von 0 % fand. In Abb. 1b,c ist das EV_HG_65 bzw. EV_HG_80 in Abhängigkeit vom 4FPTA dargestellt. In Abb. 1b zeigt sich beim Einsilberverstehen eine Spannweite von 0 bis 50 %, welche sich bei 80 dB SPL auf 0 bis 80 % vergrößert (Abb. 1c). Bei 483 (65 dB SPL) bzw. 335 (80 dB SPL) der 661 untersuchten Ohren konnte präoperativ kein Einsilberverstehen mit HG nachgewiesen werden. Die Korrelation nach Spearman (Rho) zwischen mEV sowie EV_HG_65 bzw. EV_HG_80 und dem 4FPTA ergab −0,65 (*p* < 0,001; *n* = 589; Abb. 1a), −0,39 (*p* < 0,001; *n* = 630; Abb. 1b) und −0,56 (*p* < 0,001; *n* = 625; Abb. 1c). Werden diejenigen Ohren ausgeschlossen, welche ein mEV bzw. EV mit HG von 0 % hatten, ergibt sich −0,44 (*p* < 0,001; *n* = 261; Abb. 1a), −0,33 (*p* < 0,001; *n* = 151; Abb. 1b) und −0,42 (*p* < 0,001; *n* = 293; Abb. 1c).

**Figure 2:**
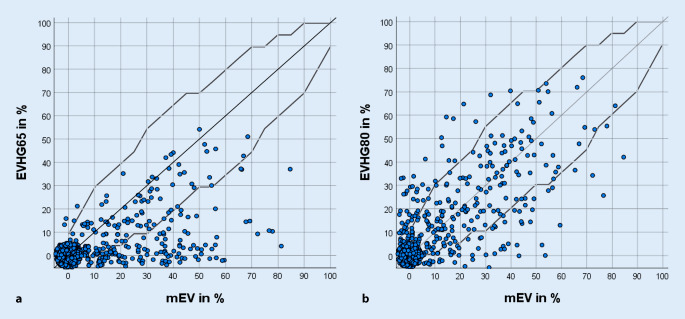

In Abb. 2 ist das EV_HG_65 und EV_HG_80 in Abhängigkeit vom mEV dargestellt. Je näher das Einsilberverstehen mit HG an der Diagonalen (graue Gerade) lag, desto besser war die Übereinstimmung mit dem mEV. Die grauen Kurven zeigen den Toleranzbereich um diese Referenzgerade, der aus dem 90%-Konfidenzintervall der Test-Retest-Genauigkeit für den Freiburger Einsilbertest bei Schwerhörigen nach Holube, Winkler und Nolte-Holube [7] definiert wurde. Für das EV_HG_80 (Abb. 2b) befinden sich mehr Daten innerhalb und oberhalb dieses Toleranzbereichs als bei 65 dB SPL (Abb. 2a). Zudem wird aus beiden Teilabbildungen ersichtlich, dass in Abb. 2a bzw. in Abb. 2b sowohl das EV_HG_65 als auch das EV_HG_80 nur in sehr wenigen Fällen aufgrund der Versorgung mit einem HG gegenüber dem mEV zunimmt.

**Figure 3:**
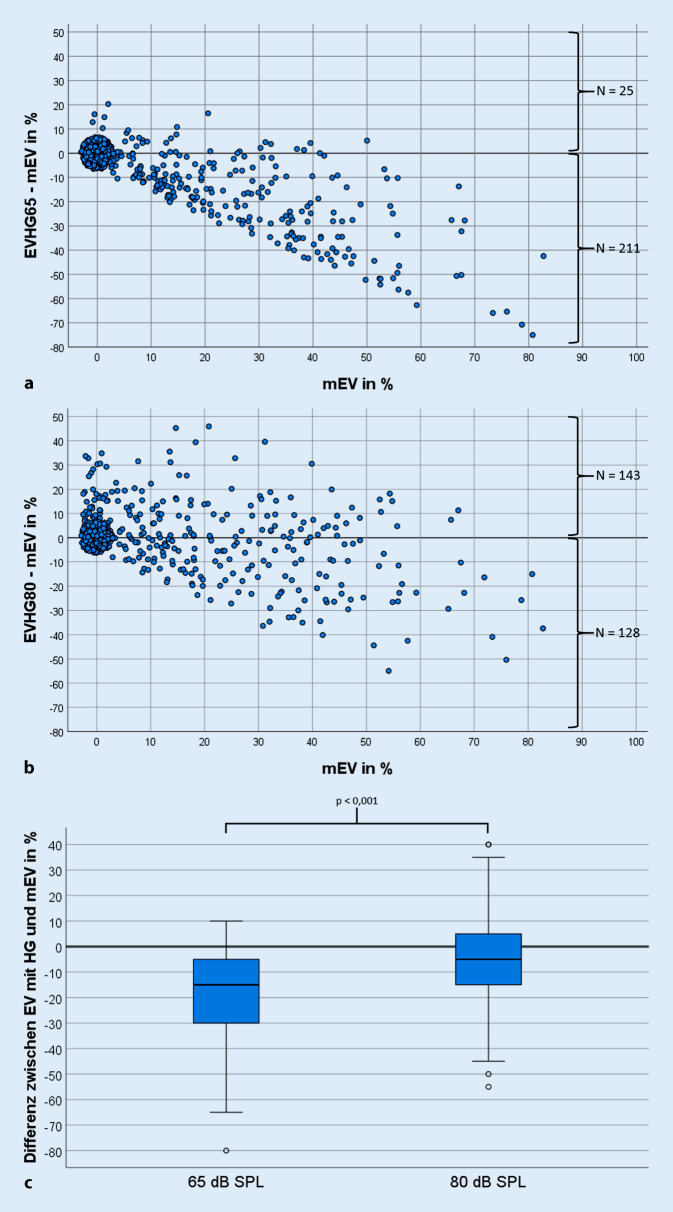

In Abb. 3 sind die Differenzen zwischen dem mEV und dem EV_HG_65 (Abb. 3a) bzw. EV_HG_80 (Abb. 3b) in Abhängigkeit vom mEV veranschaulicht. Ein Differenzwert oberhalb der grauen Referenzgeraden bei 0 % bedeutet ein besseres Verstehen mit HG im Vergleich zum mEV, und umgekehrt für Werte unterhalb dieser Geraden. Bei beiden Messpegeln konzentrieren sich die Differenzwerte im Bereich um 0 % mEV. In Abb. 3a zeigt sich mit zunehmendem mEV ein höherer Verlust des EV_HG_65 gegenüber dem mEV, der im Vergleich dazu bei 80 dB SPL schwächer ausgeprägt ist (Abb. 3b). Vielmehr verteilen sich die Differenzwerte für die Messung bei 80 dB SPL nahezu gleich um die Referenzgerade bei 0 %. Bei Messung mit 80 dB SPL wurde in 143 Fällen gegenüber dem mEV ein besseres Verstehen mit HG erreicht, bei 65 dB SPL hingegen nur in 25 Fällen.

Aufgrund der Ballung der Differenzwerte bei 0 % mEV und der daraus folgenden geringen statistischen Aussagekraft wurde weiterführend das Einsilberverstehen mit HG nur bei solchen PatientInnen analysiert, die ein mEV von mehr als 0 % aufwiesen. Die Fallzahl reduzierte sich damit von 575 (65 dB SPL, Abb. 3a) bzw. 573 Ohren (80 dB SPL, Abb. 3b) auf 247 Ohren. Für dieses korrigierte Kollektiv sind in Abb. 3c die Differenzen zwischen dem mEV und EV_HG_65 und EV_HG_80 in Form von Boxplots dargestellt. Bei dem Messpegel von 65 dB SPL liegt die mittlere Differenz bei (–18,3 ± 16,7) % und bei 80 dB SPL bei (−4,0 ± 16,4) %. Die Differenz zwischen dem Gewinn des Verstehens mit HG bei 65 und 80 dB SPL im Vergleich zum mEV (Abb. 3c) ist statistisch signifikant (*p* < 0,001).

## Diskussion

Die vorliegende Studie untersuchte die Beziehung zwischen 4FPTA, dem mEV und dem Einsilberverstehen mit HG im Rahmen der CI-Vordiagnostik. Dabei wurde das Einsilberverstehen mit HG ergänzend zum klinisch gebräuchlichen Testpegel von 65 dB SPL [1, 8, 9], welcher unter anderem repräsentativ für den Pegel von Umgangssprache ist, auch bei einem im Vergleich dazu höherem Pegel von 80 dB SPL betrachtet. Die Ergebnisse zeigen eine stärkere Übereinstimmung zwischen der tonaudiometrischen Hörschwelle und dem EV_HG_80 als zwischen Hörschwelle und EV_HG_65. Eine starke Korrelation ergab sich auch bei der Betrachtung des Einsilberverstehens mit HG bei beiden Pegeln im Vergleich zum mEV. Der Ausschluss von PatientInnen ohne substanzielle Restverständlichkeit (mEV = 0 %) zeigte die geringere Abweichung zwischen mEV und EV_HG_80 im Vergleich zum mEV und EV_HG_65 noch deutlicher auf.

Die Messung des mEV und des EV_HG_65 wird in der Hilfsmittelrichtlinie [3] als Basisdiagnostik im Rahmen der Hördiagnostik und der Erfolgskontrolle der apparativen Versorgung beschrieben und ist Gegenstand verschiedener wissenschaftlicher Veröffentlichungen [7, 17, 19]. Dabei wurde festgestellt, dass das mEV nur in wenigen Fällen mit dem HG bei 65 dB SPL erreicht werden kann und das Einsilberverstehen bei diesem Pegel oft um 20 Prozentpunkte niedriger liegt als das mEV [14]. Hoppe et al. zeigten, dass sich diese Diskrepanz oberhalb eines Lebensalters von 80 Jahren noch vergrößert [9] und die Forderung nach einer Übereinstimmung von mEV und EV_HG_65 (Differenz von < 5 % bzw. < 10 %) nur bei 23 % bzw. 40 % der Messungen erfüllt werden konnte [9]. Unsere Messungen ergaben bei identischem Prüfpegel vergleichbare Ergebnisse mit 27,5 % bzw. 46,5 % für < 5 % bzw. < 10 % Abweichung zwischen mEV und Verstehen mit HG. Bei einem Prüfpegel von 80 dB SPL hatten hingegen 61,6 % bzw. 73,3 % der Messungen eine Differenz von < 5 % bzw. < 10 %. Es zeigte sich eine mittlere Differenz von (−18 % ± 16,7 %) zwischen dem mEV und dem EV_HG_65. Die Ergebnisse dieser Arbeit befinden sich damit in Deckung mit den vorhergehenden Studien von Hoppe et al. und legen den Schluss nahe, dass sich das EV_HG_65 nur bedingt aus dem mEV vorhersagen lässt. Für einen Pegel von 80 dB SPL war die mittlere Differenz zwischen dem mEV und dem EV_HG_80 hingegen nur (−4 % ± 16,4 %) und dem angestrebten maximalen Verstehen deutlich näher als bei 65 dB SPL. Während bei leichtgradigen Schwerhörigkeiten die maximale Verbesserung im Einsilberverstehen bei niedrigen Schallpegeln auftritt [5], könnte diese Untersuchung Hinweise dafür geben, dass bei hochgradigen Schwerhörigkeiten die maximale Verbesserung im Einsilberverstehen bei Pegeln erreicht wird, die oberhalb der Pegel für Umgangssprachlautstärke liegen. Dies ist nach Engler et al. [5] auch in der Anpassung der HG begründet, welche bei hochgradigen Schwerhörigkeiten bei Umgangslautstärke mit den Hörgeräteanpassformeln NAL-NL2 und DSL v5.0 keine ausreichende Verstärkung erreichen.

Die CI-Leitlinie [1] verlangt zur Indikationsstellung im Rahmen der präoperativen CI-Diagnostik nach einem optimierten HG, welches mit seiner Einstellung ein möglichst optimales Einsilberverstehen nahe dem mEV erreichen soll. Im klinischen Alltag zeigt sich, dass dies in der Praxis aufgrund von fehlender Toleranz gegenüber der Hörgerätelautstärke oder der Verstärkung hoher Töne oft nicht erreicht wird. Es ist anzunehmen, dass das mEV nahe an der Unbehaglichkeitsschwelle gemessen wird und nur über die Kürze der sprachaudiometrischen Messung, aber nicht im Alltag toleriert wird. Eine hochgradige Schwerhörigkeit ist zwar im Alltag aufgrund des Recruitments oftmals nicht mehr adäquat mit einem HG zu versorgen, die benötigte Verstärkung ist jedoch während der Messung des Einsilberverstehens tolerierbar. Sie gibt damit im Rahmen der Diagnostik vor einer möglichen CI-Versorgung eine Aussage zur Fähigkeit der Spracherkennung unter apparativer Versorgung. In weiterführenden Untersuchungen wurde betrachtet, inwieweit das Verstehen mit HG bei 80 dB SPL zur Vorhersage des postoperativen Erfolgs mit Cochleaimplantat nutzbar ist [17]. Es konnte gezeigt werden, dass das Sprachverstehen mit Hörgerät bei 80 dB als zusätzlicher präoperativer Indikator ergänzend zum mEV für das postoperative Sprachverstehen verwendet werden kann und sollte aus diesem Grund in der klinischen Routine regelmäßig gemessen werden.

Die überwiegende Anzahl von 92 % der untersuchten PatientInnen wies einen 4FPTA von mehr als 80 dB HL auf. Mit den Hörgeräten werden dann zumeist Hörschwellen im freien Schallfeld oberhalb von 40 dB HL erzielt. Für die Gruppe der PatientInnen mit Indikation für ein Cochleaimplantat resultiert dies dann bei vielen PatientInnen bei Untersuchung mit überschwelligen Methoden, wie der 40-dBSL-Methode [15], in Sprachpegeln von > 80 dB. Dies ist aufgrund der großen Lautstärke im freien Schallfeld nicht sinnvoll durchführbar.

Die Ergebnisse der vorliegenden Arbeit legen nahe, dass im Rahmen der CI-Vordiagnostik die Ansprüche der Theorie und die Umsetzbarkeit in der Praxis eine deutliche Diskrepanz aufweisen. In der Theorie besteht der Wunsch, das vorhandene Hörvermögen optimal auszunutzen, wohingegen in der Praxis die Versorgung oft durch die technischen Möglichkeiten des HG und die Intoleranz gegenüber einer lauteren Einstellung durch die PatientInnen limitiert wird. Diese Diskrepanz ist vor allem bei hochgradigen Schwerhörigkeiten zu erkennen, bei denen die wenigen Informationen, welche noch vom Innenohr verarbeitet werden können, ausgenutzt werden müssen, um eine adäquate HG-Versorgung und damit die Verbesserung des Verstehens im Alltag zu sichern [5, 8].

Die vorliegende Studie konnte zeigen, dass die zu 65 dB SPL ergänzende Untersuchung des Einsilberverstehens mit HG bei 80 dB SPL – wie von der CI-Leitlinie empfohlen – bezogen auf hochgradige Schwerhörigkeiten bei der Erfolgskontrolle von HG sinnvoll ist, da die fehlende Verstärkung des HG durch den höheren Eingangspegel ausgeglichen werden kann. Bei hochgradigen Schwerhörigkeiten ist es deshalb empfehlenswert, das Einsilberverstehen mit HG zusätzlich zu 65 dB SPL bei einem Eingangspegel von 80 dB SPL zu messen, um das maximale mit HG erreichte Einsilberverstehen im Vergleich zum mEV nachweisen zu können.

## Fazit für die Praxis

- Das Einsilberverstehen mit Hörgerät (HG) zeigt bei einem Sprachpegel von 65 dB SPL oftmals nicht die nach Hilfsmittelrichtlinie und CI-Leitlinie geforderte Deckung mit dem mEV. Die mittlere Differenz zwischen mEV und Einsilberverstehen mit HG bei diesem Pegel beträgt (−18,3 ±16,7) %.
- Bei hochgradig schwerhörigen PatientInnen wird für das Einsilberverstehen bei 80 dB SPL mit HG eine bessere Deckung mit dem mEV erzielt, bei einer mittleren Differenz von (−4,0 ± 16,4) %. Es ist im Rahmen der klinischen Diagnostik sinnvoll, das Sprachverstehen mit HG auch bei Pegeln oberhalb von 65 dB SPL zu untersuchen. Hierfür können wir einen Sprachpegel von 80 dB SPL empfehlen.
